# Early Skewed Distribution of Total and HIV-Specific CD8^+^ T-Cell Memory Phenotypes during Primary HIV Infection Is Related to Reduced Antiviral Activity and Faster Disease Progression

**DOI:** 10.1371/journal.pone.0104235

**Published:** 2014-08-05

**Authors:** Yanina Ghiglione, Juliana Falivene, María Julia Ruiz, Natalia Laufer, María Eugenia Socías, Pedro Cahn, Luis Giavedoni, Omar Sued, María Magdalena Gherardi, Horacio Salomón, Gabriela Turk

**Affiliations:** 1 Instituto de Investigaciones Biomédicas en Retrovirus y SIDA (INBIRS), Universidad de Buenos Aires- CONICET, Buenos Aires, Argentina; 2 Fundación Huésped, Buenos Aires, Argentina; 3 Hospital J.A. Fernández, Buenos Aires, Argentina; 4 Department of Virology and Immunology, Southwest National Primate Research Center, Texas Biomedical, Research Institute, San Antonio, Texas, United States of America; Imperial College London, United Kingdom

## Abstract

The important role of the CD8^+^ T-cells on HIV control is well established. However, correlates of immune protection remain elusive. Although the importance of CD8^+^ T-cell specificity and functionality in virus control has been underscored, further unraveling the link between CD8^+^ T-cell differentiation and viral control is needed. Here, an immunophenotypic analysis (in terms of memory markers and Programmed cell death 1 (PD-1) expression) of the CD8^+^ T-cell subset found in primary HIV infection (PHI) was performed. The aim was to seek for associations with functional properties of the CD8^+^ T-cell subsets, viral control and subsequent disease progression. Also, results were compared with samples from Chronics and Elite Controllers. It was found that normal maturation of total and HIV-specific CD8^+^ T-cells into memory subsets is skewed in PHI, but not at the dramatic level observed in Chronics. Within the HIV-specific compartment, this alteration was evidenced by an accumulation of effector memory CD8^+^ T (T_EM_) cells over fully differentiated terminal effector CD8^+^ T (T_TE_) cells. Furthermore, higher proportions of total and HIV-specific CD8^+^ T_EM_ cells and higher HIV-specific T_EM_/(T_EM_+T_TE_) ratio correlated with markers of faster progression. Analysis of PD-1 expression on total and HIV-specific CD8^+^ T-cells from PHI subjects revealed not only an association with disease progression but also with skewed memory CD8^+^ T-cell differentiation. Most notably, significant direct correlations were obtained between the functional capacity of CD8^+^ T-cells to inhibit viral replication *in vitro* with higher proportions of fully-differentiated HIV-specific CD8^+^ T_TE_ cells, both at baseline and at 12 months post-infection. Thus, a relationship between preservation of CD8^+^ T-cell differentiation pathway and cell functionality was established. This report presents evidence concerning the link among CD8^+^ T-cell function, phenotype and virus control, hence supporting the instauration of early interventions to prevent irreversible immune damage.

## Introduction

Human Immunodeficiency Virus (HIV) infection causes an irreversible deterioration of the immune system ultimately leading to the development of AIDS in the vast majority of infected persons. Following virus transmission, acute/early phase of infection is characterized by high levels of peak viremia, rapid loss of CD4^+^ T-cells in both peripheral blood and mucosal lymphoid tissues, and in some cases clinical symptoms [1]. Emergence of HIV-specific CD8^+^ T-cell response is associated with the drop of plasma viremia to a stable level; known as the viral set point [2] [3]. Given the central role that HIV-specific CD8^+^ T-cells play in the control of viral replication [4], [5], special emphasis has been focused on this cell population. In order to help design an effective HIV vaccine, different parameters such as the magnitude, specificity and functionality of the CD8 response were extensively studied in different settings. Many of these works asserted that the quality of the response, rather than the quantity, might play an important role [6]–[10] [11]–[15]. Also, the phenotype of the CD8^+^ T-cell response is an important component of effective anti-viral immunity. Moreover, phenotype and function are two attributes of the response essentially linked and many research lines are currently being directed at understanding which populations along the CD8^+^ T-cell differentiation pathway are most effective in inhibiting viral replication. Recent works shed light on the complex differentiation profiles of the total and HIV-specific CD8^+^ memory T-cells and their association with antiviral function and disease progression [16]–[19].

There exists a large amount of publications reporting the characterization of HIV-specific CD8^+^ T-cells in chronic infection, however works performed during the acute infection are more limited [14], [15]. Moreover, in both cases most reports were based on subtype B or C infected cohorts rather than non-subtype B/C cohorts [16]. Our group has previously studied multiple aspects of the HIV-specific CD8^+^ T-cell subsets during acute/early HIV infection. Our findings were the first to report the immunological aspects and CD8 profile of an Argentinean cohort during the acute/early infection [20], [21]. Our last report showed that the early relative immunodominance of Gag-specific cells was associated with delayed disease progression. Also, these Gag-specific CD8^+^ T-cells had a higher capacity to degranulate, secrete IFN-γ and mediate viral inhibition activity *in vitro* (VIA). The main contribution of this study relied on the correlation between HIV-specific CD8^+^ T-cell functional properties during acute/early infection and clinical outcomes over the first year post-infection. Here, we present novel results from our ongoing work on an Argentinean cohort of recently infected subjects. As an extension of our preceding works, we aimed at performing an immunophenotypic analysis (in terms of memory markers and Programmed cell death 1 (PD-1) expression) of the CD8^+^ T-cell differentiation profile found in primary infection with viral control and subsequent disease progression. Additionally, associations between HIV-specific CD8^+^ T cell phenotype and functional properties (more specifically antiviral capacity) were studied. Evidence supporting these notions is provided.

## Materials and Methods

### Study population

A total of 63 subjects participated in this study: 10 healthy HIV-seronegative donors (HD) and 53 HIV-infected patients: 32 were enrolled during primary HIV infection (PHI), 10 were chronically infected (Chronics) and 11 were Elite Controllers (EC) (Table 1 and Table S1). PHI subjects were enrolled by the *Grupo Argentino de Seroconversión* Study Group [22] under the following inclusion criteria: (1) detection of HIV RNA or p24 antigen with a simultaneous negative or indeterminate Western Blot assay; or (2) positive Western Blot with a negative test within the previous six months. Chronically infected patients were defined as subjects with established HIV infection for over 3 years, detectable viral load (VL, >50 HIV RNA copies/ml plasma) and HAART (Highly Active Anti-Retroviral Therapy) naïve, while EC were defined as subjects infected for more than 5 years with undetectable VL (<50 HIV RNA copies/ml plasma), CD4^+^ T-cell counts >450 cells/µl blood, HAART naïve and no record of opportunistic infections and/or AIDS-related diseases. HD samples were obtained from voluntary blood donors at the *Sanatorio Dr Julio Mendez* blood bank (Buenos Aires, Argentina). All donors were >18 years; completed and passed a survey on blood donation; and were screened for serological markers before being accepted as donors.

**Table 1:** Summary of clinical data corresponding to HIV^+^ subjects enrolled per study group.

		Viral load^a^^,^ c				
Group (No. of subjects)	Median Days post-infection (IQ)	Median RNA copies/ml (IQ)	Mean log_10_ ± SD	Viral set point^d^ (mean log_10_ ± SD)	CD4^+^ T-cell count^b^^,^ c, median No. of cells/µl (IQ)	CD4 set point^d^, median No. of cells/µl (IQ)
**PHI**						
All (n = 32)	60 (30–90)	34,800 (8,843–252,588)	4.6±0.9	4.5±0.7	503 (320–682)	517 (404–629)
PHI >350 (n = 20)	60 (49–113)	18,471 (6,010–98,650)	4.3±0.9	4.3±0.6	618 (510–771)	573 (454–652)
PHI <350 (n = 12)	55 (30–90)	151,026 (34,510–500,000)	5.1±0.8	5.1±0.7	281 (230–338)	256 (158–319)
**Chronic (n = 10)**	-	28,435 (9,449–197,984)	4.5±0.7		141 (11–563)	
**EC (n = 11)**	-	<50	<1.7		602 (562–888)	

a Versant HIV-1 RNA 3.0 assay, Siemens. Lower and upper detection limits are 50 and 500,000 RNA copies/ml, respectively (1.7 and 5.7log10).

b Flow cytometry double platform, FACSCanto, BD Biosciences.

c For PHI subjects, data correspond to baseline samples. For chronic and elite controller subjects, data correspond to samples obtained at enrollment.

d Set points were not calculated for subjects that initiated HAART during the first year post-infection.

IQ: Interquartile range.

### Human samples

Blood samples were collected from study participants at enrollment, centrifuged to separate plasma, which was stored at −80°C until use. Peripheral blood mononuclear cells (PBMCs) were isolated by Ficoll-Hypaque density gradient centrifugation (GE Healthcare, Little Chalfont/Buckinghamshire, UK) and cryopreserved for subsequent phenotypic and functional assays. PBMC cryopreservation and thawing were performed following protocols developed to obtain an optimal detection of antigen specific T-cell responses [23]–[25]. For PHI subjects, samples were obtained at enrollment (baseline) and at 3, 6, 9, and 12 months post-infection. Samples used for immunophenotypic and functional analyses were obtained from subjects that remained HAART-naïve at the moment of sampling. In the case PHI subject needed to start HAART early, baseline samples were obtained before treatment instauration. Plasma VL (branched-DNA, Versant HIV-1 RNA 3.0 assay; Siemens Healthcare, Sudbury/Suffolk, UK) and CD4^+^ T-cell count (flow cytometry double platform, BD FACSCanto; BD Biosciences, San Diego/California, USA) were assessed in HIV-infected subjects.

### Human Subject Research Ethic Statement

Blood samples from HIV-infected individuals and healthy donors were obtained for this study. Prior to enrollment, the study was reviewed and approved by two institutional review boards (IRB): *Comité de Ética Humana, Facultad de Medicina, Universidad de Buenos Aires* and *Comité de Bioética, Fundación Huésped* (Buenos Aires, Argentina). Both HIV-infected participants and healthy donors provided written informed consents accepting to participate in this study.

### Peptides

Potential T-cell epitope (PTE) peptide panels corresponding to Nef, Gag and Env proteins and the CEF (cytomegalovirus -CMV- , Epstein-Barr virus, and influenza virus) peptide pool were obtained from the NIH AIDS Reagent Program (NIH, Bethesda/Maryland, USA) [26], [27]. Lyophilized peptides were dissolved in dimethyl sulfoxide (DMSO, Sigma-Aldrich, St Louis/Missouri, USA) at 40 µg/µl and stored at −20°C.

PTE peptides are 15 amino acids (a.a.) in length and contain naturally-occurring 9 a.a. sequences that are potential T-cell determinants embedded in the sequences of circulating HIV-1 strains worldwide. Here, PTE peptides were grouped in 9 pools: 1xNef (N = 127 peptides), 3xGag (corresponding to p17 (N = 97), p24 (N = 128) and p2p7p1p6 (denoted as RG, N = 95), and 5xEnv (Gp120A1 -N = 73, spans HXB2 Env a.a. positions 1–154-, Gp120A2 -N = 73, 157–284-, Gp120B -N = 105, 287–511-, Gp41A -N = 114, 513–689-, Gp41B -N = 115, 689–842-).

### Phenotypic and functional analysis of CD8^+^ T-cells by flow cytometry

**Figure 1:**
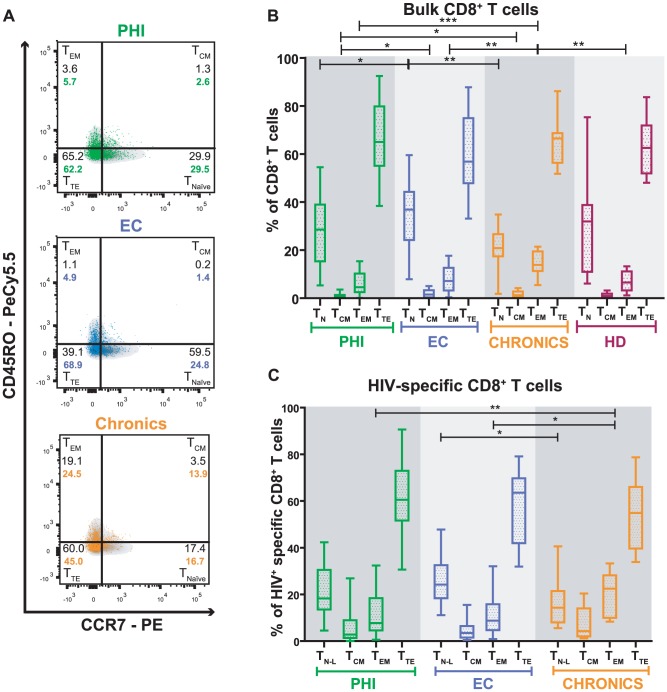
Distribution of memory sub-populations within bulk and HIV-specific CD8^+^ T-cells. (A) Density and overlay dot plots of memory subsets in total (density) and HIV-specific CD8^+^ T-cells (colored dots) of one representative subject per study group. The four defined sub-populations are identified within each quadrant: T_N_ and T_N-L_ = Naïve and Naïve-like T-cells, respectively (CCR7^+^/CD45RO^−^); T_CM_ = central memory T-cells (CCR7^+^/CD45RO^+^); T_EM_ = effector memory T-cells (CCR7^−^/CD45RO^+^); T_TE_ = terminal effectors T-cells (CCR7^−^/CD45RO^−^). Proportions of each memory subset of total (in black) and HIV-specific (in color) CD8^+^ T-cells are also shown. Panels B and C: Percentage of bulk (B) and HIV-specific (C) CD8^+^ T-cells subsets of subjects enrolled per study group. Primary HIV infection (PHI) N = 24 subjects (31 specific responses); Elite Controllers (EC) N = 11 subjects (16 specific responses); Chronics N = 10 subjects (13 specific responses); Healthy Donors (HD) N = 10. Horizontal lines stand for median values. P values were calculated using Mann-Whitney test. Asterisks denote different P values: * P<0.05; ** P<0.005; *** P<0.001.

T-cell phenotypic and functional markers were measured to identify total and HIV-specific CD8^+^ T-cell memory populations following the protocol published before [20], [21] with the following modifications: Cryopreserved PBMCs were thawed and rested overnight in RPMI medium (Sigma-Aldrich, St Louis/Missouri, USA) supplemented with 10% fetal bovine serum (Gibco, Grand Island/NY, USA), 2 mM L-glutamine (Sigma-Aldrich, St Louis/Missouri, USA), 100 U/ml penicillin (Sigma-Aldrich, St Louis/Missouri, USA), 100 mg/ml streptomycin (Sigma-Aldrich, St Louis/Missouri, USA), and 10 mM HEPES (Gibco, Grand Island/NY, USA). Cell viability was checked both immediately after thawing and after overnight rest by trypan blue exclusion. Only samples with good cell recovery (>70%) and good viability (>95%) both after thawing and resting, were used in the assays. PBMCs were dispensed in U-bottom 96-well plates (5×10^5^ cells/well) in duplicate wells. Costimulatory antibodies (anti-CD28 and anti-CD49d antibodies (1 µg/ml; BD Biosciences, San Jose/California, USA), monensin (Golgistop, 0.7 µl/ml; BD Biosciences, San Jose/California, USA), brefeldin A (10 µg/ml; BD Biosciences, San Jose/California, USA), and the corresponding peptide pool (2 µg/ml) were added. Also, a mixture of anti-CD107A-fluorescein isothiocyanate (FITC) and anti-CD107B-FITC antibodies (BD Biosciences, San Jose/California, USA) was added in each well. An unstimulated (peptide-free medium plus 0.5% DMSO and costimulatory antibodies) and two positive controls (2 µg/ml CEF peptide pool and phorbol myristate acetate (PMA)-ionomycin (Sigma-Aldrich, St Louis/Missouri, USA)) were included in each assay. Cells were incubated for 5 hours at 37°C, washed, and stained for 30 min at 37°C with anti-CCR7-phycoerythrin (PE) (BD Biosciences, San Jose/California, USA), followed by staining for 30 min at 4°C, with LIVE/DEAD Fixable NEAR-IR (Invitrogen, Life Technologies, Carlsbad/California, USA), in order to exclude dead cells, and anti-CD3, -CD8 and -CD45RO antibodies conjugated to -PECy7, -APC and -PeCy5.5 (BD Biosciences, San Jose/California, USA) respectively. For PD-1 analysis, stimulated PBMCs were surface stained with a separate panel that included anti-PD-1-PE (BD Biosciences, San Jose/California, USA), anti-CD3-PECy7, anti-CD8-APC and LIVE/DEAD Fixable NEAR-IR. Then, cells were permeabilized and fixed using the Cytofix/Cytoperm kit (BD Biosciences, San Jose/California, USA), following the manufacturer's protocol. After the permeabilization/fixation step, cells were stained intracellularly with anti-IL-2, anti-TNF-α, and anti-IFN-γ antibodies, all of them conjugated to FITC (BD Biosciences, San Jose/California, USA). Cells were then washed, resuspended in 0.5% paraformaldehyde (PFA, Merck & Co, Whitehouse station/New Jersey, USA) and stored until data acquisition in a 2-laser, 6-color BD FACSCanto flow cytometer. Data acquisition and analysis were performed using the BD FACSDiva v 6.1.3 software (BD Biosciences, San Jose/California, USA). FlowJo Vx.0.7 free trial software (FlowJo Enterprise, Treestar Inc., Ashland/Oregon, USA) was used to generate Figure 1A and Figure S1 only for illustration purposes. Instrument settings and fluorescence compensation were performed on each testing day using unstained and single-stained samples. Isotype controls, consisting of stimulated cells stained with anti-CD3 and anti-CD8 conjugated antibodies and isotype controls corresponding to phenotype (CCR7, CD45RO and PD-1) and intracellular markers, were performed for each patient in order to accurately set negative populations. First, a plot of forward scatter area (FSC-A) versus height (FSC-H) was constructed to remove doublets. Then, gating was performed on small lymphocytes in a FSC versus side scatter (SSC) plot. At least 80,000 events were acquired in the lymphocyte gate. Dead cells were then excluded on the bases of LIVE/DEAD fluorescence. Subsequently, CD3^+^ CD8^+^ cells were gated in a CD3-versus-CD8 dot plot. HIV-specific CD8^+^ T-cells were identified in a CD8 vs. cytokines (FITC) density plot (the gating strategy is provided in Figure S1). A positive cytokine response was defined as at least twice background, >0.05% after subtraction of background, and at least 50 events. This criterion was established to minimize the possibility of error due to a low number of events when further subdividing these cells into memory subsets. For phenotypic analysis, CD45RO vs. CCR7 density plot or PD-1 histogram were performed on gated CD8^+^ cells. Distribution of different phenotype subsets were analyzed both in total and HIV-specific CD8^+^ T-cell compartments.

### Immune Activation

CD4^+^ and CD8^+^ lymphocyte activation was analyzed on thawed and over-night rested PBMCs by flow cytometry. Cells were stained for 30 min at 4°C with LIVE/DEAD Fixable NEAR-IR in order to exclude dead cells, and with the following fluorochrome-conjugated antibodies (all of them obtained from BD Biosciences, San Jose/California, USA): anti-HLA-DR-FITC, anti-CD4-PerCP, anti-CD38-APC, anti-CD3-PeCy7 and anti-CD8-PE.

Data acquisition and analysis was performed using the BD FACSDiva software. Initial gating was performed on living lymphocytes followed by gating on CD3^+^CD4^+^ or CD3^+^CD8^+^ events. Isotype-matched FITC- and APC-conjugated non-specific antibodies were used in each sample in order to accurately set HLA-DR and CD38 negative populations.

### CD8^+^ T-cell Virus Inhibitory Activity (VIA) and CD8^+^ T-cell polyfuntionality

The e*x vivo* ability of CD8^+^ T-cell to inhibit viral replication in primary autologous CD4^+^ T-cells (VIA) and the capacity of HIV-specific CD8^+^ T-cells to produce cytokines (IL-2, IFN-γ and TNF- α) and degranulate (evidenced by CD107A/B mobilization) upon stimulation as well as its polyfunctionality were evaluated exactly as published elsewhere [20].

### Quantification of soluble plasma factors

Simultaneous determination of the following 39 cytokines and chemokines was performed in plasma samples from a subset of 18 PHI subjects (at baseline time-point only) using Luminex technology (MILLIPLEX MAP Human Cytokine/Chemokine, Merck Millipore, Billerica/Massachusetts, USA): EGF, Eotaxin, FGF-2, Flt-3 Ligand, Fractalkine, G-CSF, GM-CSF, GRO, IFN-α2, IFN-γ, IL-1α, IL-1β, IL-1rα, IL-2, IL-3, IL-4, IL-5, IL-6, IL-7, IL-8, IL-9, IL-10, IL-12 (p40), IL-12 (p70), IL-13, IL-15, IL-17, IP-10, MCP-1, MCP-3, MDC (CCL22), MIP-1α, MIP-1β, sCD40L, sIL-2Rα, TGF-α, TNF-α, TNF-β, VEGF. Samples were processed and analyzed as described by Giavedoni et al [28].

### Data analysis

For PHI subjects, presumed date of infection was calculated as described in [22]. Viral and CD4^+^ T-cell set-points were calculated as the geometric mean of the determinations obtained between 6 and 12 months post-presumed date of infection. Set-points were not calculated for those subjects who started HAART during the first 12 months of infection or if no stable set-point was reached during that period. Starting from pilot sample data, pre-study estimations of final sample sizes were determined using Harris, Horvitz, Mood method in order to provide 80% power, at the 5% level of significance. Statistical analyses were performed using GraphPad Prism 5 (GraphPad Software Inc., La Jolla/California, USA). All data, except log_10_ VL, were analyzed using nonparametric statistics. Two-tailed Wilcoxon and Mann-Whitney tests were used to compare intra- and inter-group variables, respectively. Correlations were determined using Spearman's rank test. All tests were considered significant if the *p* value obtained was less than 0.05.

## Results

### Study subjects description

In order to accomplish the aims of this study, three groups of HIV-infected subjects were enrolled: 32 subjects were recruited during HIV seroconversion and/or within 6 months since the presumed date of infection (PHI group), 10 chronically-infected subjects (Chronics), and 11 subjects defined as Elite Controllers (EC) according to the criteria defined in Materials and Methods. Detailed description of the HIV infected participants is shown in Table 1, Figure S2, and Table S1. Additionally, samples from 10 HIV-negative healthy donors (HD) were obtained. Baseline sample for PHI subjects were obtained at a median of 60 days post-presumed date of infection and most corresponded to Fiebig stages V and VI [29]. As regards chronically infected subjects, the individuals enrolled in this study include subjects with preserved immune status as well as subjects with advanced immune deterioration (as observed in Table S1 and Figure S2), providing evidence of the natural heterogeneity of such HIV-positive population. Additionally, immune activation was evaluated in all groups of HIV-infected subjects as it is known to be a major predictor of disease progression [30]. The percentages of CD4^+^ and CD8^+^ T-cells that expressed the immune activation markers CD38 and HLA-DR, either alone or in combination, were determined by flow cytometry. Results are shown in Figure S2C and Table 2. As expected, highest levels of immune activation were observed in baseline samples from PHIs, followed by Chronics and ECs.

**Table 2:** Immune Activation Panel corresponding to HIV^+^ subjects enrolled per study group^a^^,^ b.

	Median % (IQ)					
Group (No. of subjects)	CD4/CD38	CD4/HLA-DR	CD4/CD38/HLA-DR	CD8/CD38	CD8/HLA-DR	CD8/CD38/HLA-DR
**PHI**						
All (n = 32)	21.7 (13.8–35.1)	5.0 (1.5.–8.4)	1.4 (0.4–2.2)	44.3 (22.5–55.7)	32.4 (15.5–44.8)	15.8 (7.4–34.7)
PHI >350 (n = 20)	23.1 (13.8–33.4)	6.7 (1.5–6.4)	1.2 (0.4–2.1)	33.7 (21.3–48.5)	24.9 (11.8–39.7)	9.7 (6.0–27.8)
PHI <350 (n = 12)	18.3 (11.9–36.3)	8.2 (1.4–12.6)	1.5 (0.5–5.6)	48.2 (39.0–64.1)	40.2 (27.9–57.8)	33.4 (14.8–43.6)
**Chronic (n = 10)**	41.3 (28.1–46.1)	12.0 (5.2–18.5)	2.7 (1.1–18.5)	35.9 (24.4–91.7)	19.0 (18.2–49.8)	11.2 (5.6–24.4)
**EC (n = 11)**	22.6 (12.9–32.9)	4.7 (2.3–9.1)	0.6 (0.4–1.7)	33.5 (28.2–44.5)	17.2 (12.3–31.9)	8.2 (3.2–12.8)

a Flow cytometry double platform, FACSCanto, BD Biosciences.

b For PHI subjects, data correspond to baseline samples. For chronic and elite controller subjects, data correspond to samples obtained at enrollment.

IQ: Interquartile range.

For certain analyses, PHI subjects were further divided into two subgroups whether their CD4^+^ T-cell count dropped below 350 cells/µl at any time during the first year post-infection or not (PHI<350 and PHI>350, respectively, Table S1). By doing so, we aimed to differentiate subjects with more rapid or aggressive progression of infection (PHI<350 group) and to investigate the association of this pattern with alterations in the phenotypic distribution of CD8 subsets. The 350 cells/µl-endpoint was chosen based on the national and international recommendations for HAART initiation by the year 2010, when most of these individuals were already enrolled [22]. PHI<350 showed significantly higher VLs and lower CD4^+^ T-cell counts, both at baseline (p = 0.0321 and p<0.0001 respectively) and set-point (p = 0.0466 and p = 0.0008, respectively), compared to the PHI>350 group (Table 1).

### Distribution of memory T-cell phenotypes in total and HIV-specific CD8^+^ T-cells identified during primary HIV infection as well as viremic and aviremic chronic infections

In a previous study, we showed that CD8^+^ T-cell specificity and function were related to control of early disease progression [20]. Based on that analysis, now we aimed to investigate the distribution of memory phenotypes in both total and HIV-specific CD8^+^ T-cell compartments during primary HIV infection (using samples from 24 PHI subjects obtained at baseline, Table S1) and its association with subsequent disease progression. Also, these parameters were screened in 10 HDs, 10 Chronics and 11 ECs for comparison purposes. For this, we performed a phenotypic analysis of CD8^+^ T-cells by flow cytometry, which allowed us to define four CD8^+^ T-cell sub-populations (Figure 1A): naïve (T_Naïve_, CCR7^+^CD45RO^−^), central memory (T_CM_, CCR7^+^CD45RO^+^), effector memory (T_EM_, CCR7^−^CD45RO^+^) and terminal effector (T_TE_, CCR7^−^CD45RO^−^) cells. The distribution of these subsets was analyzed both in bulk and HIV-specific CD8^+^ T-cells. The latter was identified as cells able to degranulate (mobilize CD107A/B) and/or express cytokines (IFN-γ, IL-2 and/or TNF-α) upon stimulation with peptide pools corresponding to Nef, p24, p17 or p2p7p1p6 proteins. All these molecules (CD107A/B, IFN-γ, IL-2 and TNF-α) were identified using antibodies stained with the same fluorochrome (FITC) in order to detect all cells responsive to stimulation, regardless of their specific functionality. Figure S1 illustrates the gating strategy used.

#### i. Distribution of memory subsets within bulk CD8^+^ T-cell is abnormal in viremic Chronics but does not significantly distinguish PHI subjects, ECs or HDs

The distribution of memory phenotypes within the total CD8^+^ T-cell compartment showed the following hierarchy in all groups of subjects analyzed: T_TE_>T_Naïve_>T_EM_>T_CM_ (Figure 1B). Although this hierarchy was conserved in all groups, differences in the proportion of the various sub-populations among groups were observed. The highest median proportion of total CD8^+^ T_Naïve_ cells (as defined for the purposes of this work) was found in ECs (36.9%; IQ25–75: 24.2–44.2), followed by HDs (31.9%; IQ25–75: 11.0–38.8), PHI subjects (29.0%; IQ25–75: 15.4–38.9) and Chronics (20.9%; IQ25–75: 17.4–26.6). The difference was not statistically significant between ECs and HDs, it barely reached statistical significance between ECs and PHI subjects (p = 0.0493) and differed significantly in ECs versus Chronics (p = 0.0018). This is in line with a previous report that indicates that naïve T-cells still comprise a large proportion of the T-cell compartment at early times post-infection [19]. On the other hand, the highest proportion of total CD8^+^ T_TE_ cells was observed in Chronics (66.5%; IQ25–75: 56.4–68.7), followed by PHI subjects (64.2%; IQ25–75: 55.3–79.5), HDs (62.6%; IQ25–75: 51.9–71.8) and ECs (56.8%; IQ25–75: 47.9–74.9) (Figure 1B). The median proportion of total CD8^+^ T_CM_ cells was statistically lower in PHI subjects (0.6%; IQ25–75: 0.2–1.3) compared both to Chronics (1.3%; IQ25–75: 0.6–2.8; p = 0.0127) and ECs (1.5%; IQ25–75: 0.3–3.3; p = 0.0405). Finally, it was observed that Chronics had a statistically higher proportion (≈2-fold) of total CD8^+^ T_EM_ cells (13.9%; IQ25–75: 11.3–19.5), compared to ECs (7.1%; IQ25–75: 3.2–13.0; p = 0.0048), HDs (6.5%; IQ25–75: 3.2–11.2; p = 0.0076) and PHI subjects (4.5%; IQ25–75: 2.5–10.1; p<0.0001). When a similar analysis was performed within PHI subgroups (PHI>350 versus PHI<350) no statistically significant difference was observed. Overall, these results indicate that the distribution of memory phenotypes within the total CD8^+^ T-cell compartment evaluated at early time-points post-infection is similar to that of HDs and ECs. In contrast, a higher proportion of the most differentiated T_EM_ and T_TE_ cells was found in viremic Chronics while a higher proportion of the less differentiated T_Naïve_ and T_CM_ cells (similar to the scenario observed in HDs) was found in aviremic chronically-infected subjects (i.e. ECs) (Figure 1B).

#### ii. HIV-specific CD8^+^ T-cells from recently infected subjects preserve a maturation hierarchy similar to that of ECs, whereas it is skewed in chronically viremic infected subjects

The distribution of memory phenotypes within the HIV-specific CD8^+^ T-cell compartment mostly mirrored that of the total compartment. However, important differences were also observed. It is worth noting here that, due to technical constraints, our definition of naïve T-cells is not strict enough to completely avoid inclusion of very early differentiated memory cells such as the so called stem-cell memory T-cells. To completely avoid this problem, additional surface markers should be included in the panel [31]. Hence, when referring to HIV-specific CCR7^+^CD45RO^−^ CD8^+^ T-cells, we used the term “naïve-like” (T_naïve-like_).

**Figure 2:**
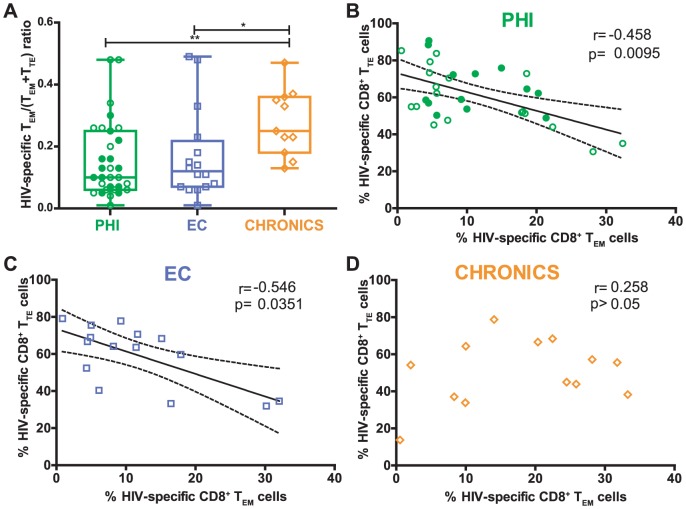
Arrest of HIV-specific CD8^+^ T-cells from effector memory (T_EM_) to terminal effector (T_TE_) of subjects enrolled. HIV-specific T_EM_/(T_EM_+T_TE_) ratio (A) of subjects enrolled per study group. Correlation among HIV-specific CD8^+^ T_TE_ and the T_EM_ cells in primary HIV infection (PHI) (B), elite controllers (EC) (C) and Chronic (D) groups. PHI group N = 24 subjects (31 specific responses); EC N = 11 subjects (16 specific responses); Chronics N = 10 subjects (13 specific responses). Panels A and B, open and filled green dots denote PHI>350 and PHI<350 subjects, respectively. Panel A, P values were calculated using Mann-Whitney test. Asterisks denote different P values: * P<0.05; ** P<0.005. Panels B–D, r and P values correspond to Spearman's test.

The highest proportion of HIV-specific CD8^+^ T_naïve-like_ cells was observed in ECs (24.2%; IQ25–75: 18.4–32.5) followed by PHI subjects (18.5%; IQ25–75: 14.0–33.3) and Chronics (14.34%; IQ25–75: 8.0–21.4; p = 0.0164) (Figure 1C). Chronics had a significantly higher proportion of HIV-specific CD8^+^ T_EM_ cells (22.5%; IQ25–75: 10.0–28.2), compared to PHI subjects (7.4%; IQ25–75: 4.6–18.3; p = 0.002) and ECs (8.8%; IQ25–75: 4.7–15.8; p = 0.0111) and tended to have a lower proportion of HIV-specific CD8^+^ T_TE_ cells (54.9%; IQ25–75: 39.7–66.0) than PHI subjects (58.9%; IQ25–75: 51.3–72.9) and ECs (63.5%; IQ25–75: 41.9–69.8). This is in line with previous studies which indicate that, during chronic progressive HIV infection, there is a maturation arrest of HIV-specific CD8^+^ T-cells from T_EM_ to T_TE_ [6], [32]. To provide further insights into this notion, the HIV-specific T_EM_/(T_EM_+T_TE_) ratio was analyzed in all groups in order to picture the proportion of T_EM_ cells out of the total of the most differentiated subsets from our panel (T_EM_ plus T_TE_). In consonance with the hypothesis mentioned above, the T_EM_/(T_EM_+T_TE_) ratio was significantly higher in Chronics compared to ECs (p = 0.0144) and PHI (p = 0.0042) (Figure 2A). No statistically significant difference was observed between ECs and PHIs or within PHI subgroups (PHI>350 versus PHI<350). Moreover, the percentages of HIV-specific CD8^+^ T_EM_ and T_TE_ cells negatively correlated in PHI subjects (r = −0.458; p = 0.0095; Figure 2B) and ECs (r = −0.546; p = 0.0351; Figure 2C) whereas no correlation at all was observed in Chronics (r = 0.258; p>0.05; Figure 2D). These results support the notion that differentiation of T_TE_ cells from T_EM_ cells is altered in viremic chronic HIV-1 infection. However, it might not be the case in ECs or, more importantly, in recently infected subjects (PHI group) where these results indicate that there exists an early preservation of the CD8^+^ T-cell compartment before going into the chronic stage of infection.

Finally, based on previous results indicating that Gag-specific CD8^+^ T-cells improved antiviral functions [20], it was hypothesized that the distribution of memory phenotypes within the HIV-specific CD8^+^ compartment would vary according to the antigens used as stimulus; i.e. Nef versus Gag (p24, p17 or RG) peptide pools. However, no clear association was found between antigen specificity and CD8^+^ T-cell memory/effector phenotype in either group analyzed (data not shown).

### Baseline higher proportions of total and HIV-specific CD8^+^ T_EM_ cells as well as higher HIV-specific T_EM_/(T_EM_+T_TE_) ratio correlated with markers of faster progression while higher proportions of T_naive_ and T_naive-like_ CD8^+^ T-cells associated with markers of slower disease progression

According to the data described above on the distribution of memory phenotypes within both total and HIV-specific CD8^+^ T-cell compartment in groups with differential disease outcome, together with data collected from the bibliography, it was hypothesized that within the PHI group, the relative frequency of the different memory subsets evaluated at baseline would be associated with disease progression. Thus, the relative frequency of a given subset measured at early time-points post-infection could be postulated as an indicator of subsequent disease progression rate. To test this, correlation analyses were performed between the percentages of the different memory subsets and the clinical data of the subjects enrolled, obtained at baseline and during the first year post-infection.

**Figure 3:**
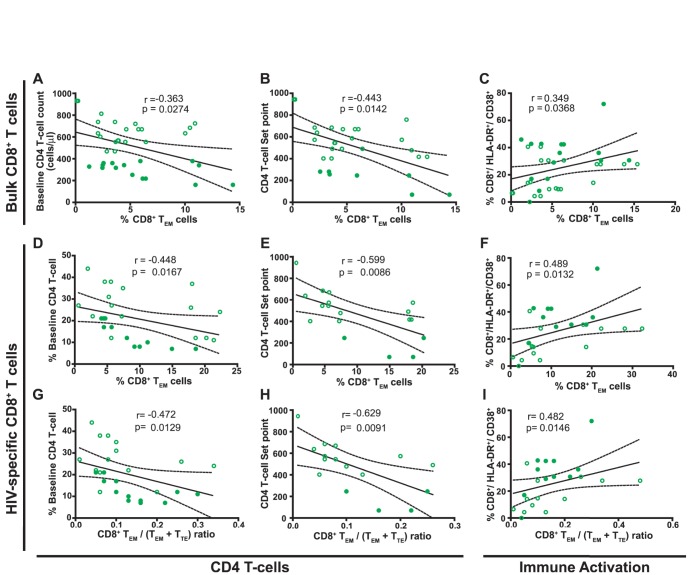
Correlations between the proportion of CD8^+^ T_EM_ cells within bulk (A–C) and HIV-specific (D–F) compartments as well as the HIV-specific CD8^+^ T_EM_/(T_EM_+T_TE_) ratio (G–I) with clinical parameters measured in baseline samples from primary HIV infected (PHI) subjects. Baseline CD4 T-cell counts (A), immune set point (B) and baseline immune activation (C) versus proportion of bulk CD8^+^ effector memory (T_EM_) cells. Percentage of baseline CD4 T-cell (D), immune set point (E) and baseline immune activation (F) versus proportion of HIV-specific CD8^+^ T_EM_ cells. Percentage of baseline CD4 T-cell (G), immune set point (H) and baseline immune activation (I) versus HIV-specific CD8^+^ T_EM_/(T_EM_+T_TE_) ratio. PHI group N = 24 subjects (39 responses analyzed for bulk compartment and 31 responses for the specific compartment). For set point correlations N = 15 subjects. In all panels, open and filled green dots denote PHI>350 and PHI<350 subjects, respectively. All r and P values correspond to Spearman's test.

As regards the total CD8^+^ T-cell compartment, and always within the PHI group, the proportion of T_EM_ cells correlated inversely with baseline CD4^+^ T-cell count (r = −0.363; p = 0.0274; Figure 3A) and directly with baseline activation levels within the CD8^+^ T-cell compartment (r = 0.349; p = 0.0368; Figure 3C). Moreover, the proportion of T_EM_ cells also correlated negatively with the CD4^+^ T-cell count set-point (r = −0.443; p = 0.0142; Figure 3B) indicating that the early proportion of this subset is associated with the subsequent level of immune set-point. When analyzing the HIV-specific CD8^+^ T-cells compartment it was found that the proportion of HIV-specific T_EM_ cells inversely correlated with baseline CD4^+^ T-cells (r = −0.448; p = 0.0167, Figure 3D) and the CD4^+^ T-cell count set-point (r = −0.599; p = 0.0086, Figure 3E). The same significant trends were observed when the HIV-specific T_EM_/(T_EM_+T_TE_) ratio was measured (r = −0.472; p = 0.0129, Figure 3G; r = −0.629; p = 0.0091, Figure 3H, respectively). Additionally, the proportion of HIV-specific T_EM_ cells directly correlated with viral set-point (r = 0.571; p = 0.0208, see Figure S3F). Then, the CD8^+^ T-cell activation was analyzed. Direct correlations both with the percentage of HIV-specific T_EM_ cells (r = 0.489; p = 0.0132, Figure 3F), and with the HIV-specific T_EM_/(T_EM_+T_TE_) ratio (r = 0.482; p = 0.0146, Figure 3I) were found. Furthermore, the HIV-specific T_EM_/(T_EM_+T_TE_) ratio was also found to directly correlate with baseline plasma levels of cytokines associated with disease progression such as IP-10 (r = 0.499; p = 0.0251), IL-1a (r = 0.509; p = 0.0220), and IL-15 (r = 0.538; p = 0.0144, not shown). On the other hand, the proportion of bulk CD8^+^ T_Naïve_ cells correlated directly with baseline CD4^+^ T-cell count (r = 0.330; p = 0.0432, Figure S3A) and inversely with baseline CD4^+^ T-cell activation (r = −0.542; p = 0.0008; Figure S3B). Similarly, the proportion of T_naïve-like_ cells directly correlated with the percentage of baseline CD4^+^ T-cells (r = 0.383; p = 0.0368, Figure S3C) while it inversely correlated with baseline VL (r = −0.460; p = 0.0093, Figure S3D) and viral set-point (r = −0.630; p = 0.0022, Figure S3E).

Overall, in line with the inter-group analysis performed above, these results indicate that a higher relative proportion of both total and HIV-specific CD8^+^ T_Naïve_ (or T_naïve-like_) cells during early time-points post-infection correlated with better immune status in terms of CD4^+^ T-cell count, at baseline and set-point. Conversely, higher proportions of the more differentiated T_EM_ and its accumulation relative to terminally differentiated cells within the HIV-specific compartment (evaluated as the HIV-specific T_EM_/(T_EM_+T_TE_) ratio) correlated with markers of faster disease progression: lower baseline and set-point CD4^+^ T-cell counts, higher viral set-point (only for the HIV-specific subset), and higher baseline levels of cellular and soluble markers of immune activation.

### PD-1 expression on CD8^+^ T-cells during PHI is related to disease progression and also with memory CD8^+^ T-cell differentiation

**Figure 4:**
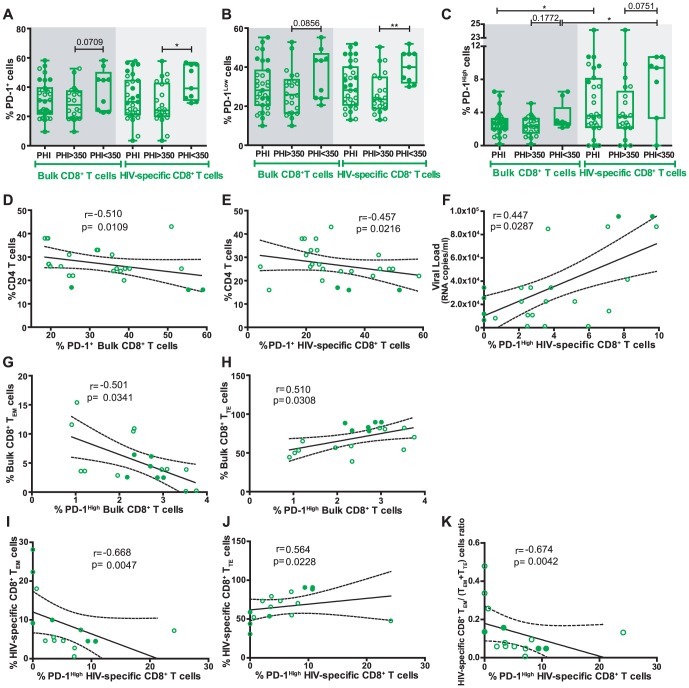
The relationship between PD-1 expression and markers of disease progression as well as with the distribution of memory subsets were studied in primary HIV infection (PHI) group. Percentage of PD-1 (A), PD-1^Low^ (B), and PD-1^High^ (C) cells out of bulk and HIV-specific CD8^+^ T-cells, in samples from PHI subjects (N = 19; 30 specific responses) obtained at 8 months post-infection. (D to F) Correlations between clinical parameters and percentage of PD-1 expression in the CD8^+^ T-cell subset, within the PHI group: Percentage of CD4 T-cells versus percentage of bulk PD-1^+^ CD8^+^ T-cells (D) or PD-1^+^ HIV-specific CD8^+^ T-cells (E). Viral load versus percentage of PD-1^High^ HIV-specific CD8^+^ T-cells (F). (G to K) Correlation between baseline CD8^+^ T-cell memory subsets and percentage of PD-1 expression at 8 months post-infection (N = 11 subjects; 18 specific responses): percentage of bulk CD8^+^ effector memory (T_EM_) (G) or terminal effector (T_TE_) (H) cells versus percentage of bulk PD-1^High^ CD8^+^ T-cells. Percentage of HIV-specific CD8^+^ T_EM_ (I), CD8^+^ T_TE_ (J) cells, or T_EM_/(T_EM_+T_TE_) ratio (K) versus percentage of PD-1^High^ HIV-specific CD8^+^ T-cells. Panels A–C: horizontal lines stand for median values. P values were calculated using Mann-Whitney test. Asterisks denote different P values: * P<0.05; ** P<0.005; *** P<0.001. Panels D–K: r and P values correspond to Spearman's test. In all panels, open and filled green dots denote PHI>350 and PHI<350 subjects, respectively.

In order to provide more insights into the phenotype of CD8^+^ T-cells found in HIV infection, the expression of Programmed cell death 1 (PD-1), a molecule commonly linked to immune exhaustion, was evaluated on bulk and HIV-specific CD8^+^ T-cells from a subset of 19 PHI subjects using samples obtained at 8±1 months post-infection (due to cell sample availability). The relationship between PD-1 expression and markers of disease progression as well as with the distribution of memory subsets were studied in this group. First, it was observed that PHI<350 subjects displayed higher proportions of both total and HIV-specific PD-1^+^ CD8^+^ T-cells, compared to PHI>350 (45.6% versus 28.8%, p = 0.0709 and 40% versus 25%, p = 0.0109, respectively; Figure 4A). Despite not reaching statistical significance, a clear tendency was observed. This trend was also recorded when PD-1^+^ events were subdivided into PD-1^low^ (Figure 4B) and PD-1^high^ (Figure 4C) phenotypes according to the intensity (on the basis of mean fluorescence intensity) of PD-1 expression. Interestingly, bulk and HIV-specific CD8^+^ T-cells only differed significantly regarding the proportion of PD-1^high^ cells (p = 0.0294, Figure 4C). In this sense, strong positive correlations were observed between bulk and HIV-specific compartments regarding the proportion of total PD-1^+^ cells (r = 0.7008, p<0.0001) and PD-1^low^ cells (r = 0.7674, p<0.0001, not shown). However, no significant correlation was found in PD-1^high^ cells (r = 0.3308, p = 0.07, not shown). This indicates that PD-1 is preferentially up-regulated in HIV-specific cells as described elsewhere [33]–[35]. Also, regarding specific cells, no PD-1 expression difference was observed in cells with different specificities (i.e. when Nef, Gag of CEF pools were used as stimuli). According to the difference observed between PHI>350 and PHI<350 subjects, negative correlations were found between both bulk and HIV-specific PD-1^+^ CD8^+^ T-cells and CD4^+^ T-cell percentages (r = −0.510, p = 0.0109 and r = −0.457, p = 0.0216, respectively, Figures 4D and 4E). Otherwise, no other association between PD-1 expression (measured as %PD-1 or PD-1 MFI) on CD8^+^ T-cells (either total or HIV-specific) and markers of disease progression (viral load, viral set-point, soluble or cellular immune activation markers) was found. Alternatively, when analyzing the proportion of PD-1^low^ and PD-1^high^ phenotypes, a direct correlation between PD-1^high^ CD8^+^ T-cells and viral load was found (r = 0.447, p = 0.0287, Figure 4F).

Then, we sought to investigate the relationship between the pattern of PD-1 expression and the distribution of memory subsets during primary HIV infection. It was found that the percentages of PD-1^high^ CD8^+^ T-cells, both within bulk and HIV-specific compartments, negatively correlated with the proportion of bulk and HIV-specific CD8^+^ T_EM_ cells (r = −0.501, p = 0.0341 and r = −0.668, p = 0.0047, respectively; Figures 4G and 4I). Conversely, positive correlations were observed with the proportion of bulk and HIV-specific CD8^+^ T_TE_ cells (r = −0.510, p = 0.0308 and r = −0.564, p = 0.0228, respectively; Figures 4H and 4J). Additionally, HIV-specific T_EM_/(T_EM_+T_TE_) ratio also correlated inversely with the proportion of PD-1^high^ CD8^+^ T-cells (r = −0.674, p = 0.0042; Figure 4K). This is in consonance with the notion that PD-1 is not only a marker of immune cell exhaustion but also its expression is related to CD8^+^ T-cell differentiation stage and activation status [36].

### CD8^+^ T-cell antiviral activity is related to CD8^+^ T-cell memory differentiation but not to PD-1 expression during primary HIV infection

**Figure 5:**
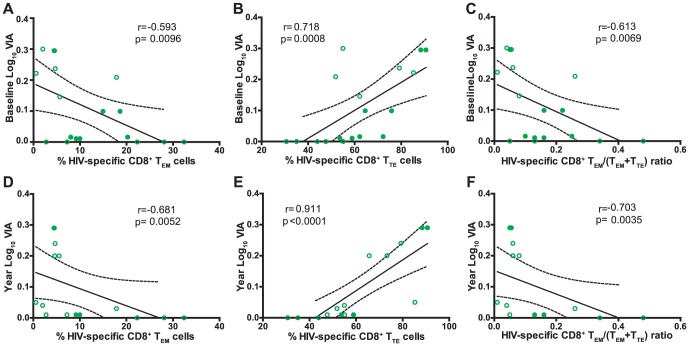
Correlation between CD8^+^ T-cell capacity to suppress HIV replication *ex vivo* (VIA) and percentage of baseline HIV-specific CD8^+^ T-cell subsets: CD8^+^ T-cell antiviral capacity measured at baseline versus percentage of HIV-specific CD8^+^ effector memory (T_EM_) cells (A), CD8^+^ terminal effector (T_TE_) cells (B), or CD8^+^ T_EM_/(T_EM_+T_TE_) ratio (C). Antiviral CD8^+^ T-cell capacity measured at 12-months post-infection versus percentage of baseline HIV-specific CD8^+^ T_EM_ cells (D), CD8^+^ T_TE_ cells (E), or CD8^+^ T_EM_/(T_EM_+T_TE_) ratio (F). N =  11 subjects (18 specific responses). In all panels, open and filled green dots denote PHI>350 and PHI<350 subjects, respectively. All r and P values correspond to Spearman's test. VIA: Viral inhibitory activity.

We have previously shown that higher HIV-specific CD8^+^ T-cell antiviral capacity during primary HIV infection was associated with higher CD4^+^ T-cell counts at set-point [20]. Thus, we sought to investigate the relationship between CD8^+^ T-cell phenotype and functionality in a subset of 11 PHI subjects from our cohort. This subset of 11 PHI subjects remained HAART-naïve during the study period. In the first place, no association was found between the memory phenotype and the capacity of specific CD8^+^ T-cell to exert a particular function (degranulate or secrete cytokines or chemokines) or with the proportion of polyfunctional CD8^+^ T-cells. Additionally, no association was found among the expression of PD-1 by CD8^+^ T-cells and its functionality. Contrarily, significant inverse correlations were observed between CD8^+^ T-cell antiviral activity (VIA) evaluated at baseline with the concurrent proportion of HIV-specific CD8^+^ T_EM_ cells (r = −0.593, p = 0.0096; Figure 5A) and the HIV-specific CD8^+^ T_EM_/(T_EM_+T_TE_) ratio (r = −0.613, p = 0.0069; Figure 5C). Conversely, a positive correlation was obtained with the proportion of HIV-specific CD8^+^ T_TE_ cells (r = 0.718, p = 0.0008; Figure 5B). This result indicates that the magnitude of CD8^+^ T-cell antiviral activity during recent infection is related to a higher proportion of HIV-specific cells with a fully differentiated phenotype, rapidly able to exert effector functions. Concomitantly, a higher level of CD8^+^ T-cell differentiation arrest (evidenced by higher T_EM_/(T_EM_+T_TE_) ratios) translates into lower CD8^+^ T-cell antiviral activity. Moreover, identical correlations were obtained when analyzing CD8^+^ T-cell phenotype at baseline and antiviral activity at 12 months post-infection (Figure 5D to 5F) indicating that the early CD8^+^ T-cell differentiation hierarchy is also associated with CD8^+^ T-cell antiviral function beyond the establishment of the set-point.

## Discussion

Data accumulated over the last years have established that the HIV-specific CD8^+^ T-cell response plays a critical role in viral control (reviewed in [4], [5], [37]). For this reason, efforts have been made to understand the properties of CD8^+^ T-cells (in terms of function and/or phenotype) that best correlate with control of viral replication [5]. Moreover, this information will be instrumental for developing and enhancing immunization strategies focused on eliciting appropriate immune responses as well as for defining currently-lacking immune correlates of protection in order to evaluate the performance of vaccine candidates. In line with this, research studies on primary HIV infection (PHI) are increasing worldwide to better understand the natural history of HIV infection and to identify the early pathogenic events that may set the course for subsequent disease progression. More specifically, cohort studies addressing the association of particular features of CD8^+^ T-cell responses arising during acute/early HIV infection with potential markers associated with disease progression are fundamental. However, most of these studies were performed in developed countries, and scarce information exists from other settings, such as South America, where local studies are needed to comprehend particular characteristics of the infection. It is worth highlighting that the capacity of cohort studies to provide meaningful contributions relies on the definition of rigorous inclusion criteria, which in turn allows for powerful comparisons of both intra- and inter-studies. In this line, our group studied multiple aspects of the HIV-specific CD8^+^ T-cell subset (specificity, *ex vivo* viral inhibitory capacity and polyfunctionality) arising early after infection, in a well-defined cohort of acute/early infected subjects from Argentina, in comparison with that found in, also local, viremic Chronics and ECs [20]. As an extension of that preceding work, here we aimed at performing immunophenotypic analyses (in terms of memory markers and PD-1 expression) of the CD8^+^ T-cells, using the same cohorts of subjects, in order to seek for associations with both CD8^+^ T-cell functional properties and with viral control and subsequent disease progression. Major findings indicate that i) the distribution of total and HIV-specific CD8^+^ T-cell memory subsets is severely altered in chronically infected subjects (excluding ECs). Although it is also altered in recently infected subjects (PHI group), the shift is not so profound as in Chronics (Figures 1 and 2); ii) within the PHI group, higher proportions of T_naive_ and T_naive-like_ CD8^+^ T-cells associated with markers of slower disease progression while higher proportions of total and HIV-specific CD8^+^ T_EM_ cells as well as higher HIV-specific T_EM_/(T_EM_+T_TE_) ratio correlated with markers of faster progression (Figures 3 and S3); iii) analysis of PD-1 expression on total and HIV-specific CD8^+^ T-cells from PHI subjects revealed an association not only with disease progression but also with memory CD8^+^ T-cell differentiation (Figure 4); and iv) hierarchy of memory CD8^+^ T-cell subsets correlated with CD8^+^ T-cell activity during primary HIV infection (Figure 5). Of note, we consider that this study represents an important extension beyond the scope of previous studies on CD8^+^ T-cell phenotype during HIV infection, focused only on acute/early infection, ECs or in comparing two opposite study populations (for instance, ECs versus rapid progressors). Here, we simultaneously studied three groups of HIV-infected subjects at different disease stages (acute versus chronic) and with different clinical outcomes (controlled versus non-controlled). Moreover, clinical follow-up of subjects identified during primary infection allowed us to correlate the CD8^+^ T-cell phenotype observed at baseline with subsequent disease progression. In addition, we simultaneously studied cell phenotype both in bulk and HIV-specific CD8^+^ T-cells, contrary to other studies focused on the total compartment only. Finally, a relationship between the distribution of CD8^+^ T-cell phenotypes and CD8^+^ T-cell antiviral function could be established, a field in which only a few reports exist (see below).

So far, several groups have studied the distribution of memory CD8^+^ subsets in HIV infection. The use of different markers and the different numbers of such markers included in each study make it a difficult task to compare and interpret inter-study results. With some discrepancies, the general picture indicates that the hallmark of HIV infection in terms of memory CD8^+^ T-cell subsets consists in an accumulation of not fully differentiated cells. This phenomenon was first described as a block toward terminal differentiation within the HIV-specific compartment in progressive chronic HIV infection [38] and subsequently confirmed even in other settings such as acute infection [6], [34], [39]. Interestingly enough, many studies have established a link between HIV-specific memory CD8^+^ T-cell differentiation and disease progression [16], [40], [41]. Additionally, differentiation of total CD8^+^ T-cells is also skewed in HIV infection and related to progression [6], [16], [19], [42], [43]. In this context, our results reinforce the level of knowledge into this field providing further support into these notions. In the first place, and as reported elsewhere [16], [19], our results indicate that the distribution of memory subsets within bulk CD8^+^ T-cell is abnormal in viremic Chronics but does not significantly distinguish PHI subjects, ECs or HDs. Similarly, the HIV-specific CD8^+^ T-cell compartment was only severely compromised in Chronics but not in the PHI group. In this scenario, early HAART initiation has been shown to provide not only virological but also immunological benefits to HIV-infected subjects. For instance, it was shown that the normal hierarchy in CD8^+^ T-cell subset differentiation is not restored after HAART-driven viral suppression [43]–[45] and that very early HAART initiation limits the seeding of the HIV reservoir, particularly in long-lived T_CM_ cells [46], [47]. Altogether, these data argue in favor of early initiation of HAART, in order to prevent irreversible deterioration of the mechanisms involved in immune homeostasis. Further studies involving the relationship between immune preservation (in terms of function and phenotype), size and features of viral reservoir together with disease progression in a PHI cohort are guaranteed.

Intriguingly, it was observed that the memory differentiation hierarchy between the total and HIV-specific compartments did not differ significantly in either group analyzed. This might reflect that the force driving CD8^+^ T-cell differentiation in HIV infection equally affects both compartments. Also, the memory differentiation pattern did not differ between Gag-specific versus Nef-specific responses, as previously reported [41], [48]. Contrary to this, Meyer-Olson *et al.* [39] described that epitope-specific CD8^+^ T-cell maturation into memory/effector phenotypes is a TCR-dependent process. The absence of difference in the specificities observed in our study may be associated with the use of peptide pools as stimuli which may mask such single-epitope differences.

As stated above, HIV infection is characterized by an accumulation of preterminally differentiated (CD45RO^+^/CCR7^−^ or T_EM_ as defined for the purposes of this work) HIV-specific CD8^+^ T-cells and relative diminished frequency of fully differentiated effector cells (CD45RO^−^/CCR7^−^ or T_TE_). Our inter-group analysis supports this notion since Chronics had a significantly higher proportion of HIV-specific CD8^+^ T_EM_ cells than PHI subjects and ECs (p = 0.002 and p = 0.0111, respectevily) and a trend to a lower proportion of HIV-specific CD8^+^ T_TE_. Most important, Chronics had significantly higher HIV-specific T_EM_/(T_EM_+T_TE_) ratios reflecting the accumulation of T_EM_ over T_TE_ in Chronics but not in ECs (Chronics vs ECs p = 0.0144) or PHI (Chronics vs PHI p = 0.0042). The same could be observed in the correlation analysis of proportions of T_EM_ versus T_TE_ in all groups of subjects: significant inverse correlations were obtained for PHI (p = 0.0095) (as in [40]) and ECs (p = 0.0351) but not for Chronics. Even more notably, the correlation analysis performed within the PHI group indicated that the HIV-specific T_EM_/(T_EM_+T_TE_) ratio evaluated at baseline inversely correlated with baseline and set-point CD4^+^ T-cell levels and directly with cellular and soluble markers of immune activation. This result, which adds support to previously reported findings in other cohorts [16], [40], clearly indicate that the early deterioration of CD8^+^ T-cell differentiation pathway associates with disease progression even at very early times post-infection. In this sense, our results, among many other reports [16], [44], [49], have suggested a direct link between maturation misbalance of the CD8^+^ T-cell memory compartment and the generalized and persistent immune activation found in HIV^+^ subjects. However, which is the cause and which the consequence is still not fully understood. A recent study focused on ECs [50] suggested that, in these particular subjects, maintenance of a less mature memory CD4^+^ T-cell population provides the necessary T-cell help for optimal maturation of effective CD8^+^ T-cell responses. In other words, the skewed memory CD8^+^ T-cell phenotype might be the result of improper CD4^+^ T-cell help. Alternatively, it was proposed that higher proportions of T_Naïve_ cells in EC may reflect increased thymic output in these subjects (compared to Chronics) which would contribute to the replenishing of such compartment [51]. Similar investigations in primary HIV infection are guaranteed to elucidate the impact of memory CD4^+^ T-cell differentiation as well as thymic function on the CD8^+^ T-cell subset in the context of progressive HIV infection.

Expression of PD-1 on virus-specific CD8^+^ T-cells has been consistently associated with a state of cellular exhaustion in the context of persistent viral infections, with HIV infection not being an exception [33], [52]–[54]. However, data on acute HIV infection is much more scarce [16], [34], [43], [55]. Here, the expression of PD-1 was evaluated in bulk and HIV-specific CD8^+^ T-cells at 8 months post-infection and related to clinical outcome as well as memory CD8^+^ T-cell differentiation and functionality, aiming to provide a broader picture of CD8^+^ T-cell phenotype hallmark in primary HIV infection. In the first place, it was found that PD-1^+^ CD8^+^ T-cells (both total and HIV-specific) were augmented in the PHI<350 group, which had significantly lower CD4^+^ T-cell counts compared to the PHI>350 group (p = 0.0341 and p = 0.0308, respectively), suggesting a relationship of PD-1 expression with faster disease progression. In line with this observation, inverse correlations were obtained for PD-1^+^ CD8^+^ T-cells and CD4^+^ T-cell counts. Similarly, other reports on primary HIV infection [16], [34] (and contrary to that described for chronic infection [33], [53]), found no associations between total PD-1 expression and viral load. However, a relationship between the magnitude of PD-1 expression and viral replication became evident when total PD-1^+^ cells were split into PD-1^Low^ and PD-1^High^ cells: higher proportions of PD-1^High^ cells directly correlated with higher viral load. This is consistent with the notion that PD-1 is up-regulated on CD8^+^ T-cells due to T-cell activation in the presence of high viral loads [36]. In this line, there is an increasing body of evidence suggesting that PD-1 might be linked to T-cell exhaustion and also, it would be a marker of cell activation [16], [36] and a key regulator of memory cell differentiation [35], [43], [56] and survival [57]. Our results indicate that, at early times post-primary infection (8 months post infection), higher proportions of PD-1^high^ CD8^+^ T-cells correlated with lower CD8^+^ T_EM_ and higher CD8^+^ T_TE_ proportions. Likewise, other authors indicated that PD-1 is expressed in all memory subsets, and that PD-1 up-regulation is associated with cellular activation, with a reduction in proliferative potential and with a higher sensitivity to cell death [16], [36], [43], [53], [56], [57]. Moreover, these results provide further support to the revisited idea that, during acute infections, PD-1 is a cellular activation marker rather than an exhaustion marker as it is in chronic infections [43], [58]. Also in this context, several reports have demonstrated that PD-1^+^ CD8^+^ T-cells can be fully functional [34], [58]–[60]. This is consistent with our failure in identifying associations between PD-1 expression and HIV-specific CD8^+^ T-cell functionality during primary HIV infection, as Petrovas *et al*. [57] reported for chronic infection.

Contrary to what was obtained for PD-1 expression, significant associations were found between memory CD8^+^ T-cell phenotype and functionality. More precisely, lower and higher baseline proportions of HIV-specific T_EM_ and T_TE_ CD8^+^ T-cells, respectively, correlated with higher *ex vivo* CD8^+^ T-cell antiviral activity (VIA), both at baseline (p = 0.0096 and p = 0.0008, respectively) and at 12 months post-infection (p = 0.0052 and p<0.0001, respectively). Antiviral activity as evaluated in this work encompasses both lytic and non-lytic antiviral mechanisms, evidencing the overall capacity of HIV-specific cells to mediate virus control. Previous reports have demonstrated, by isolating pure populations of CD8^+^ T-cells (based on their memory differentiation) from chronically HIV-infected subjects and vaccinees, that cells from all subsets could mediate VIA [15], [61], [62]. In these reports, Elite Controller status [62] and control of breakthrough infections in vaccinated monkeys [61] were associated with improved antiviral activity of the CD8^+^ T_EM_ compartment. In this line, here we were able to find a relationship between the hierarchy of memory CD8^+^ T-cell differentiation and CD8^+^ T-cell antiviral function in primary infection. Even when *a priori* these results seem to be in contradiction, it must be noted that the experimental approach and, most important, the setting are completely different: we evaluated CD8^+^ T-cell antiviral function in the context of high viral load and immune activation such as acute/early infection meanwhile CD8^+^ T-cells from ECs and vaccines are not subjected to such a hostile environment affecting its functionality. This raises concerns over whether signatures of CD8^+^ T-cell function or phenotype found in individuals such as ECs are the cause or the consequence of virus control. Many reports argue in favor of the latter hypothesis so caution should be taken when interpreting such data. Conversely to VIA, no association was found between memory CD8^+^ T-cell differentiation and polyfunctionality. This is in contradiction with a report by Riou *et al.* [41] showing that HIV-specific CD8^+^ T-cells show decreasing polyfunctionality coinciding with an increase in differentiation from early to terminally differentiated memory subsets during HIV acute/early infection. Due to technical constraints, these differences may be masked by our experimental design.

Overall, here we report that normal maturation of total and HIV-specific CD8^+^ T-cells into memory subsets is skewed in PHI but not at the dramatic level observed in chronic infection. Furthermore, the magnitude of this alteration in maturation translates into a decrease in CD8^+^ T-cell antiviral capacity which is directly correlated with early disease progression. Unscrambling relationships among T-cell differentiation, T-cell functionality, immune activation, viral control, and disease progression in multiple settings (primary infection, viremic Chronics and ECs), such as those observed in this work, are increasingly important to advance our understanding of HIV pathogenesis. As well, this information will be instrumental for therapeutic and sterilizing vaccine design in order to boost our ability to elicit beneficial responses.

## Supporting Information

Figure S1 **Gating strategy used for the identification of different CD8 sub-populations, based on their phenotype, on bulk and HIV-specific T-cells.** Illustration data were derived from one representative subject, stimulated with an HIV peptide pool. Initial gating was performed on a plot of forward scatter area (FSC-A) versus height (FSC-H) to remove doublets. Then, gating was performed on small lymphocytes in a plot of forward scatter (FSC) versus side scatter (SSC). Dead cells were then excluded on the basis of LIVE/DEAD fluorescence. Subsequently, CD3^+^ CD8^+^ cells were gated in a CD3-versus-CD8 dot plot. Following identification of these cells, HIV-specific CD8^+^ T-cells were identified in a CD8 versus. cytokines (FITC) density plot. Then, the distribution of different phenotype subsets were analyzed both in total and HIV-specific CD8^+^ T-cell compartments. For this, CD45RO versus CCR7 density plots or PD-1 histograms (also PD-1^Low^ or PD-1^High^) were performed on gated CD3^+^CD8^+^ cells (bulk) or CD8^+^ Cytokines^+^ cells (HIV-specific). Simultaneous use of CD45RO and CCR7 markers allowed us to define four CD8^+^ T-cell sub-populations: naïve (T_Naïve_, CCR7^+^CD45RO^−^), central memory (T_CM_, CCR7^+^CD45RO^+^), effector memory (T_EM_, CCR7^−^CD45RO^+^) and terminal effector (T_TE_, CCR7^−^CD45RO^−^) cells.
(EPS)

Figure S2 **Three groups of HIV infected subjects were enrolled for this study: 32 subjects were recruited during HIV seroconversion and/or within 6 months since the presumed date of infection (PHI group), 10 chronically infected subjects (Chronics), and 11 subjects defined as Elite Controllers (EC) according to the criteria defined in** **materials and methods** **.** Viral load (A) CD4^+^ T-cell count (B) and Immune Activation (C) were determined. Panels A and B, values corresponding to both baseline and set point samples are shown for Primary HIV infected (PHI) subjects. Viral and CD4^+^ T-cell set-points were calculated as the geometric mean of determinations obtained between 6 and 12 months post-presumed date of infection. Also, subjects included in either PHI>350 and PHI<350 subgroups (defined in materials and methods) are indicated by open and filled green dots, respectively. Horizontal lines stand for median values. P values were calculated using Mann-Whitney test. Asterisks denote different P values: * P<0.05; ** P<0.005; *** P<0.001. Within the PHI group, median baseline VL and CD4+ T-cell counts were 34,800 RNA copies/ml (interquartile range (IQ)25–75: 8,843–252,588 copies/ml) and 503 cells/µl (IQ25–75: 320–682 cells/µl), respectively. As regards chronically infected subjects, median VL was 28,435 RNA copies/ml (IQ25–75: 9,449–197,984) and median CD4+ T-cell count was 141 cells/µl (IQ25–75: 11–563) which was significantly lower than the other groups (p = 0.016 and p = 0.0028 compared to PHI and ECs, respectively). On the other hand, all ECs had undetectable plasma VL (<50 RNA copies/ml) and the median CD4^+^ T-cell count was 602 cells/µl (IQ25–75: 562–888). PHI<350 showed, both at baseline and set-point, significantly higher VLs (p = 0.0321 and p<0.0001, respectively) and lower CD4^+^ T-cell counts (p = 0.0466 and p = 0.0008, respectively), compared to the PHI>350 group and (see also Table 1).
(EPS)

Figure S3 **Correlations between the proportion of the different CD8^+^ T-cell subsets within bulk (A and B) and the HIV-specific compartment (C to F) and clinical parameters measured in baseline samples from primary HIV infected (PHI) subjects: Baseline CD4^+^ T-cell counts (A) and baseline CD4 immune activation (B) versus percentage of CD8^+^ T_Naive_ cells.** Percentage of HIV-specific CD8^+^ T_Naive-like_ cells versus percentage of baseline CD4^+^ T-cell (C), baseline viral load (D) and viral set-point (E). (F) Percentage of HIV-specific CD8^+^ T_EM_ cells versus viral set-point. PHI group N = 24 subjects (39 responses analyzed for bulk compartment and 31 responses for the specific compartment). For set point correlations N = 15 subjects. In all panels, open and filled green dots denote PHI>350 and PHI<350 subjects, respectively. All r and P values correspond to Spearman's test.
(EPS)

Table S1 Characteristics of HIV^+^ subjects enrolled per study group.
(DOCX)
