# Supplementary material for: Early Skewed Distribution of Total and HIV-Specific CD8+ T-Cell Memory Phenotypes during Primary HIV Infection Is Related to Reduced Antiviral Activity and Faster Disease Progression
Source: PLoS One. 2014 Aug 5;9(8):e104235. doi: 10.1371/journal.pone.0104235 (PMC4122399; doi:10.1371/journal.pone.0104235)
Supplement: Table S1 — Characteristics of HIV+ subjects enrolled per study group. (DOCX) [file pone.0104235.s004.docx]

| **Table S1:** Characteristics of HIV^+^ subjects enrolled per study group. | | | | | | | | | | | |
| --- | --- | --- | --- | --- | --- | --- | --- | --- | --- | --- | --- |
|  |  |  |  |  | Viral Load (VL)^2^ | |  |  |  |  |  |
| Patient ID | Sex | Age (years) | Infection stage^1^ | Days post infection | RNA copies/ml | Log_10_ | Viral Set point^3^ Log_10_ | CD4^+^ T cell count^2^ Cells/μl | CD4 set point^3^ Cells/μl | CD4<350 cells/μl during first year^3, 4^ | HAART^5^ |
| **Primary HIV Infection (PHI) N=32** | | | | | | | | | | | |
| NP01* | F | 26 | VI | 60 | 17420 | 4,2 | 4,1 | 685 | 402 | NO | NO |
| NP02* | F | 27 | VI | 60 | 116129 | 5,1 | 4,8 | 671 | 477 | NO | NO |
| NP03* | F | 37 | V | 75 | 9532 | 4,0 | 4,0 | 590 | 587 | NO | NO |
| NP04* | F | 34 | V | 30 | >500000 | >5,7 | 4,3 | 603 | 490 | NO | NO |
| NP05* | F | 43 | VI | 60 | 258015 | 5,4 | 4,5 | 803 | 670 | NO | NO |
| NP06* | F | 29 | V | 30 | 3265 | 3,5 | NA | 440 | NA | NO | NO |
| NP07* | M | 73 | VI | 120 | 13962 | 4,1 | 4,6 | 577 | 544 | NO | NO |
| NP08* | M | 47 | V | 60 | 104 | 2,0 | 2,8 | 602 | 638,5 | NO | NO |
| NP09 | M | 43 | V | 30 | 19522 | 4,3 | 4,2 | 525 | 442 | NO | NO |
| NP10 | M | 28 | VI | 60 | 37169 | 4,6 | 4,6 | 505 | 411 | NO | NO |
| NP11* | M | 26 | V | 90 | 1093 | 3,0 | 4,2 | 1012 | 417 | NO | NO |
| NP12* | M | 40 | VI | 60 | 8613 | 3,9 | 4,4 | 778 | 758 | NO | NO |
| NP13* | M | 32 | VI | 150 | 256051 | 5,4 | 4,8 | 797 | 943 | NO | NO |
| NP14* | M | 39 | VI | 150 | 46211 | 4,7 | 4,8 | 633 | 573 | NO | NO |
| NP15* | M | 25 | V | 30 | 32918 | 4,5 | 4,5 | 748 | 568 | NO | NO |
| NP16* | M | 30 | V | 45 | 455417 | 5,7 | 5,2 | 698 | 685 | NO | NO |
| NP17 | M | 54 | V | 90 | 2707 | 3,4 | 3,9 | 902 | 600 | NO | NO |
| NP18 | M | 41 | VI | 120 | 32473 | 4,5 | 4,7 | 500 | 652 | NO | NO |
| NP19 | M | 32 | V | 170 | 5142 | 3,7 | 3,1 | 499 | 581 | NO | NO |
| NP20 | M | 28 | V | 60 | 12736 | 4,1 | 5,1 | 490 | 454 | NO | NO |
| P01* | F | 38 | VI | 150 | 199754 | 5,3 | NA | 252 | NA | YES | YES |
| P02* | F | 47 | IV | 30 | >500000 | >5,7 | 5,7 | 379 | 246 | YES | YES |
| P03* | F | 41 | VI | 90 | 3662 | 3,6 | 3,9 | 302 | 281 | YES | NO |
| P04* | F | 28 | VI | 30 | 102297 | 5,0 | NA | 222 | NA | YES | YES |
| P05* | M | 26 | VI | 30 | 35378 | 4,5 | NA | 317 | NA | YES | YES |
| P06 | M | 42 | VI | 150 | 34221 | 4,5 | 5,2 | 399 | 356 | YES | YES |
| P07* | M | 25 | VI | 60 | 242199 | 5,4 | 5,6 | 161 | 70 | YES | YES |
| P08* | M | 25 | V | 90 | 7643 | 3,9 | NA | 328 | NA | YES | YES |
| P09* | M | 26 | VI | 30 | 4182153 | 6,6 | NA | 104 | NA | YES | YES |
| P10* | M | 43 | V | 50 | 98684 | 5,0 | 4,9 | 256 | 256 | YES | NO |
| P11 | M | 45 | IV | 60 | >500000 | >5,7 | NA | 259 | NA | YES | YES |
| P12* | M | 45 | V | 30 | >500000 | >5,7 | NA | 341 | NA | YES | YES |
| **Chronics (C) N=10** | | | | | | | | | | | |
| C01 | F | 38 | Chronic | - | 22475 | 4,4 | - | 143 | - | - | NO |
| C02 | F | 29 | Chronic | - | 14784 | 4,2 | - | 139 | - | - | NO |
| C03 | F | 45 | Chronic | - | 36399 | 4,6 | - | 555 | - | - | NO |
| C04 | M | 45 | Chronic | - | 4718 | 3,7 | - | 441 | - | - | NO |
| C05 | M | 24 | Chronic | - | 2889 | 3,5 | - | 606 | - | - | NO |
| C06 | M | 27 | Chronic | - | 34395 | 4,5 | - | 4 | - | - | NO |
| C07 | M | 35 | Chronic | - | 291184 | 5,5 | - | 13 | - | - | NO |
| C08 | M | 40 | Chronic | - | 179591 | 5,3 | - | 16 | - | - | NO |
| C09 | F | 24 | Chronic | - | 253164 | 5,4 | - | 5 | - | - | NO |
| C10 | M | 47 | Chronic | - | 11026 | 4,0 | - | 585 | - | - | NO |
| **Elite controllers (EC) N=11** | | | | | | | | | | | |
| EC01 | F | 43 | Chronic | - | <50 | <1,7 | - | 817 | - | - | NO |
| EC02 | M | 44 | Chronic | - | <50 | <1,7 | - | 549 | - | - | NO |
| EC03 | F | 48 | Chronic | - | <50 | <1,7 | - | 1261 | - | - | NO |
| EC04 | F | 36 | Chronic | - | <50 | <1,7 | - | 562 | - | - | NO |
| EC05 | F | 49 | Chronic | - | <50 | <1,7 | - | 602 | - | - | NO |
| EC06 | F | 33 | Chronic | - | <50 | <1,7 | - | 888 | - | - | NO |
| EC07 | M | 36 | Chronic | - | <50 | <1,7 | - | 456 | - | - | NO |
| EC08 | M | 39 | Chronic | - | <50 | <1,7 | - | 595 | - | - | NO |
| EC09 | F | 56 | Chronic | - | <50 | <1,7 | - | 612 | - | - | NO |
| EC10 | F | 60 | Chronic | - | <50 | <1,7 | - | 570 | - | - | NO |
| EC11 | F | 38 | Chronic | - | <50 | <1,7 | - | 888 | - | - | NO |
| ^1.^ PHI subjects were stratified according to Fiebig stages (Fiebig et al. 2003 AIDS, 17:1871-9). ^2.^ For PHI subjects, data correspond to baseline samples. For Chronic and EC subjects, data correspond to samples obtained at enrollment. ^3.^ Only applicable to PHI subjects. ^4.^ States if CD4^+^ T-cell count dropped below 350 cells/μl at any time during the first year post-infection, thus defining PHI<350 and PHI>350 subgroups. ^5.^ Regarding PHI subjects, data illustrates if the subjects started HAART during the first year post-infection. For Chronics and ECs, data represents if subjects ever received HAART. * Denote PHI subjects where memory phenotype analysis was performed. F: Female. M: Male. NA: Data not available. | | | | | | | | | | | |
